# Effects of multimodal nursing intervention with health education on recovery, pain, and psychological outcomes after laparoscopic cholecystectomy: a retrospective cohort study

**DOI:** 10.3389/fsurg.2025.1708726

**Published:** 2026-01-14

**Authors:** Zhongfang Hu, Huan Liu

**Affiliations:** 1 Department of Nursing, Fuzhou Municipal Hospital, Fuzhou, Jiangxi, China; 2 Department of Stomatology, Fuzhou Maternal and Child Health Hospital, Fuzhou, Jiangxi, China

**Keywords:** laparoscopic cholecystectomy, multimodal nursing intervention, health education, postoperative recovery, pain management, immune function

## Abstract

**Objective:**

To explore the effects of multimodal nursing intervention combined with health education on postoperative recovery, pain management, and psychological state in patients undergoing laparoscopic cholecystectomy.

**Methods:**

The clinical data of 100 patients who underwent laparoscopic cholecystectomy in our hospital from January 2023 to December 2024 were retrospectively analyzed. The patients were divided into a control group (CG, *n* = 50, routine care) and an intervention group (IG, *n* = 50, multimodal nursing intervention combined with health education) according to the nursing method. The two groups were compared in terms of postoperative recovery indicators, pain scores, psychological state, immune function indicators, incidence of complications, and functional recovery scores.

**Results:**

The IG demonstrated significantly shorter time to first flatus, first defecation, first ambulation, hospital stay, and incision healing than the CG (*P* < 0.05). VAS scores were significantly lower in the IG at all postoperative time points (*P* < 0.05). SAS and SDS scores were significantly lower in the IG at discharge (*P* < 0.05). The IG displayed higher CD4+ level than the CG at discharge (*P* < 0.05). The IG exhibited significantly lower incidence of abdominal distension and total incidence of postoperative complications than the CG (*P* < 0.05). The scores of daily activities, self-care ability, pain control, and overall recovery in the IG were significantly higher than those in the CG (*P* < 0.05).

**Conclusion:**

Multimodal nursing intervention combined with health education can significantly promote postoperative recovery of patients undergoing laparoscopic cholecystectomy, effectively control postoperative pain, improve psychological state and immune function, reduce the incidence of complications, and improve the quality of functional recovery.

## Introduction

Since its introduction in the late 1980s, laparoscopic cholecystectomy (LC) has become the preferred surgical procedure for the treatment of benign gallbladder diseases due to its advantages such as minimal trauma, rapid recovery, and short hospital stay (1). According to statistics, approximately 750,000 LC surgeries are performed globally each year, with a postoperative complication rate of 3%–15% (2). However, clinical practice indicates that despite the minimally invasive nature of LC, patients still face many challenges after surgery, including postoperative pain, gastrointestinal dysfunction, and psychological stress reactions, which not only affect the patient's recovery process, but may also lead to decreased immune function and increase the risk of complications (3, 4). The traditional nursing model mainly focuses on the disease itself and basic nursing operations, and lacks holistic attention to the patient's multi-dimensional physiological, psychological and social needs (5). In recent years, with the promotion of the concept of enhanced recovery after surgery (ERAS) and the development of nursing disciplines, multimodal nursing intervention has gradually gained attention as a comprehensive nursing strategy. This model emphasizes the organic integration of multiple nursing measures to provide comprehensive care for patients from multiple dimensions, such as pain management, early mobilization, nutritional support, and psychological intervention, to achieve optimal recovery outcomes (6, 7).

As a core component of modern nursing, health education can improve patients’ disease awareness, enhance their self-management ability, and improve treatment compliance by providing systematic and individualized health information. Studies (8) have shown that adequate health education can not only relieve patients’ anxiety, but also increase their initiative to participate in rehabilitation, thereby improving clinical outcomes. It is worth noting that the impact of surgical trauma on the body's immune function has received increasing attention. Although LC is a minimally invasive surgery, it can still cause a certain degree of immunosuppression, manifested as an imbalance in the proportion of T lymphocyte subsets and a decrease in cellular immune function (9). The degree of recovery of immune function is directly related to the patient's anti-infection ability and overall recovery effect. Therefore, it is of great significance to explore effective nursing intervention measures to promote the recovery of immune function.

To date, no study has comprehensively evaluated the effects of multimodal nursing intervention combined with health education on immune function in LC patients. Although previous research has demonstrated the role of multimodal nursing in postoperative recovery (10), there are few systematic evaluations of its impact on immune function, especially the lack of comprehensive evaluation of key immune markers such as CD4+ T lymphocytes. In addition, previous studies have mostly been small-sample or single-dimensional assessments, lacking systematic evaluations of multiple dimensions such as pain, psychology, immunity and functional recovery. This study aims to systematically evaluate the comprehensive effect of multimodal nursing combined with health education on improving the clinical outcomes of patients with LC by innovatively conducting multidimensional evaluations, with particular emphasis on the regulatory effect on perioperative immune indicators, so as to provide evidence-based support for optimizing nursing programs for LC patients. Therefore, this study retrospectively analyzed the clinical data of 100 LC patients and comprehensively evaluated the application effect of this nursing model from multiple dimensions such as postoperative recovery, pain management, psychological state, and immune function, aiming to provide an evidence-based basis for optimizing the nursing plan for LC patients.

## Research methods

### Research design

This is a retrospective cohort study, reviewed and approved by the ethics committee of Fuzhou Maternal and Child Health Hospital. The study protocol conforms to the ethical requirements of the Declaration of Helsinki. The study was carried out following the STROBE guidelines. Given the retrospective nature of the study, informed consent was waived with the approval of the ethics committee.

### Research subjects

Patients who underwent LC in the Department of General Surgery of our hospital between January 2023 and December 2024 were screened through the hospital information system according to the following inclusion and exclusion criteria. A retrospective study design was selected for the following reasons: (1) The nursing model has already been routinely implemented in our hospital, and prospective studies have ethical constraints; (2) Retrospective analysis enables rapid analysis of nursing outcomes, providing a foundation for subsequent prospective research; (3) Complete medical records and standardized data collection procedures ensure data quality.

#### Inclusion criteria

(1) Age 18–75 years; (2) Diagnosed with benign diseases such as gallstones and gallbladder polyps by abdominal ultrasound, CT or MRCP; (3) First time undergoing elective LC; (4) American Society of Anesthesiologists (ASA) grade I–II; (5) Normal preoperative liver and kidney function, complete blood count, and coagulation function; (6) Complete medical records, including perioperative immune function indicators; (7) Postoperative hospital stay ≥72 h.

#### Exclusion criteria

(1) Emergency surgery such as acute suppurative cholecystitis and gallbladder perforation; (2) Conversion to laparotomy during surgery; (3) Combined with malignant tumors; (4) Previous history of upper abdominal surgery; (5) Combined with severe cardiovascular and cerebrovascular diseases, liver and kidney dysfunction; (6) Long-term use of immunosuppressants or hormone drugs; (7) History of cognitive dysfunction or mental illness; (8) Severe complications after surgery requiring transfer to the ICU; (9) Perioperative blood transfusion recipients; (10) Incomplete medical records or missing key data.

### Sample size calculation

Based on preliminary pilot results, the expected difference in time to first postoperative flatus between the two groups was approximately 6 h, with a standard deviation of about 8 h. With *α* = 0.05 (two-tailed) and *β* = 0.20 (80% test power), according to the sample size calculation formula of the independent samples t-test, at least 45 patients were needed in each group. Considering a 10% data loss rate, the final sample size was determined to be 50 patients in each group, with a total of 100 patients.

### Grouping method

According to the nursing intervention measures, the patients were divided into IG and CG. To minimize selection bias, patients were grouped based on admission time. Patients admitted from January 2023 to December 2023 were categorized into the CG (receiving routine care during this period), while those admitted from January 2024 to December 2024 were categorized into the IG (receiving multimodal care during this period). The surgical teams, anesthesia protocols, and postoperative basic treatment regimens were identical for both patient groups, with only the nursing approach differing.

The CG (*n* = 50) received routine care, including routine preoperative preparation and basic health education, postoperative vital sign monitoring (once every 4–6 h), medication as prescribed by the clinicians and observation of drug reactions, basic life care and wound care, routine dietary guidance (fasting from food and water for 6 h postoperatively, followed by gradually resuming diet), encouragement of appropriate activities for patients, and routine guidance before discharge.

In addition to routine care, patients in the IG received the following multimodal nursing interventions. (1) Multimodal pain management, which included administering analgesics 30 min before surgery; dynamically adjusting the analgesic regimen based on the patient's pain score; and also implementing non-drug analgesic measures such as music therapy, progressive muscle relaxation, acupoint massage, and cold compresses according to the patient's condition (11, 12). (2) Psychological nursing intervention. This included psychological assessment and counseling before surgery; cognitive behavioral intervention to correct patients’ misconceptions; establishing therapeutic communication with patients and providing emotional support; and guiding patients to practice relaxation techniques such as deep breathing and meditation (13). (3) Early mobilization plan. Patients were instructed to start passive exercises in bed 2 h after surgery, assisted in semi-recumbent position 6 h after surgery, and encouraged to ambulate out of bed 12 h after surgery. (4) Nutritional management. Patients were allowed to drink small amounts of warm water 2 h after surgery and start a liquid diet 6 h after surgery according to the recovery of gastrointestinal function. (5) Immune function support. Patients were ensured adequate daily sleep and were provided with appropriate protein and vitamin supplements when necessary. (6) Systematic health education. One-on-one explanations were provided to patients, covering disease-related knowledge, surgical procedures, postoperative recovery points, complication recognition and prevention measures, etc. Follow-up method: All patients completed relevant assessments at discharge. A portion of patients underwent telephone follow-up one week after discharge to assess their recovery.

### Outcome measures

#### Baseline data

Age, sex, body mass index (BMI), operative time, intraoperative blood loss, etc.

#### Main outcome measures

(1) Postoperative recovery indicators: time to first flatus, time to first defecation, time to first ambulation, length of hospital stay (LOS), and incision healing time. (2) Pain score: The visual analog scale (VAS, 0–10 points) was used to assess the pain intensity at 6 h, 12 h, 24 h, 48 h, and 72 h after surgery. (3) Psychological status: The Self-Rating Anxiety Scale (14) (SAS, standard score ≥50 points for anxiety) and the Self-Rating Depression Scale (15) (SDS, standard score ≥53 points for depression) were used to assess the psychological status before surgery and at discharge.

#### Secondary outcome measures

(1) Immune function indicators: peripheral blood T lymphocyte subsets (CD3+, CD4+, CD8+) and CD4+/CD8+ ratio were measured at admission and discharge by flow cytometry. (2) Postoperative complications: nausea and vomiting, abdominal distension, incision infection, bile leakage, etc. (3) Functional recovery score: The functional recovery scale was used to assess daily activities, self-care ability, pain control, and overall recovery at discharge. Each item was scored from 0 to 100, with higher scores indicating better recovery.

### Data collection and quality control

Data were collected independently by two trained researchers using standardized data collection forms. Data were entered by two researchers and cross-checked. Data with discrepancies were adjudicated by a third researcher. The VAS scores were performed by the same group of nurses to ensure consistent assessment standards.

### Statistical methods

The data collected in this study were statistically analyzed using SPSS 26.0. The continuous data were found to be normally distributed by the Shapiro–Wilk test and expressed as mean ± standard deviation (mean ± SD). The independent sample t-test was used for inter-group comparison. The categorical data were expressed as percentages, and the chi-square test was used for inter-group comparison. *P* < 0.05 was considered statistically significant. To control for potential confounding factors, variables with significant baseline differences (if present) were adjusted using analysis of covariance (ANCOVA). *post-hoc* power analysis revealed that based on the actual sample size and observed effect size, the statistical power for testing the primary outcome measures in this study exceeded 80%.

## Results

### Comparison of baseline clinical data between the two groups of patients

The baseline clinical data of the two groups of patients were included and compared between the groups. The results showed that there was no statistically significant difference between the two groups in terms of average age, sex distribution, BMI index, operative time, etc. (*P* > 0.05), indicating that the two groups were comparable, providing a solid foundation for subsequent outcome analyses (Table 1).

**Table 1:** Comparison of baseline clinical data between the two groups of patients (mean ± SD)/ [*n* (%)].

Clinical data	CG (*n* = 50)	IG (*n* = 50)	*t/χ* ^2^	*P*
Age (years)	48.32 ± 12.47	47.86 ± 11.83	0.165	0.869
Sex (male/female)	22/28	24/26	0.163	0.686
BMI (kg/m^2^)	24.13 ± 3.18	24.52 ± 3.41	0.605	0.547
Operative time (min)	42.76 ± 15.28	43.51 ± 14.69	0.234	0.816
Intraoperative blood loss (mL)	15.24 ± 8.37	14.82 ± 7.91	0.245	0.807
Comorbidities (yes/no)	18/32	16/34	0.178	0.673
Educational level (high school or above/below high school)	35/15	37/13	0.271	0.603

### Comparison of postoperative recovery indicators between the two groups

**Figure 1:**
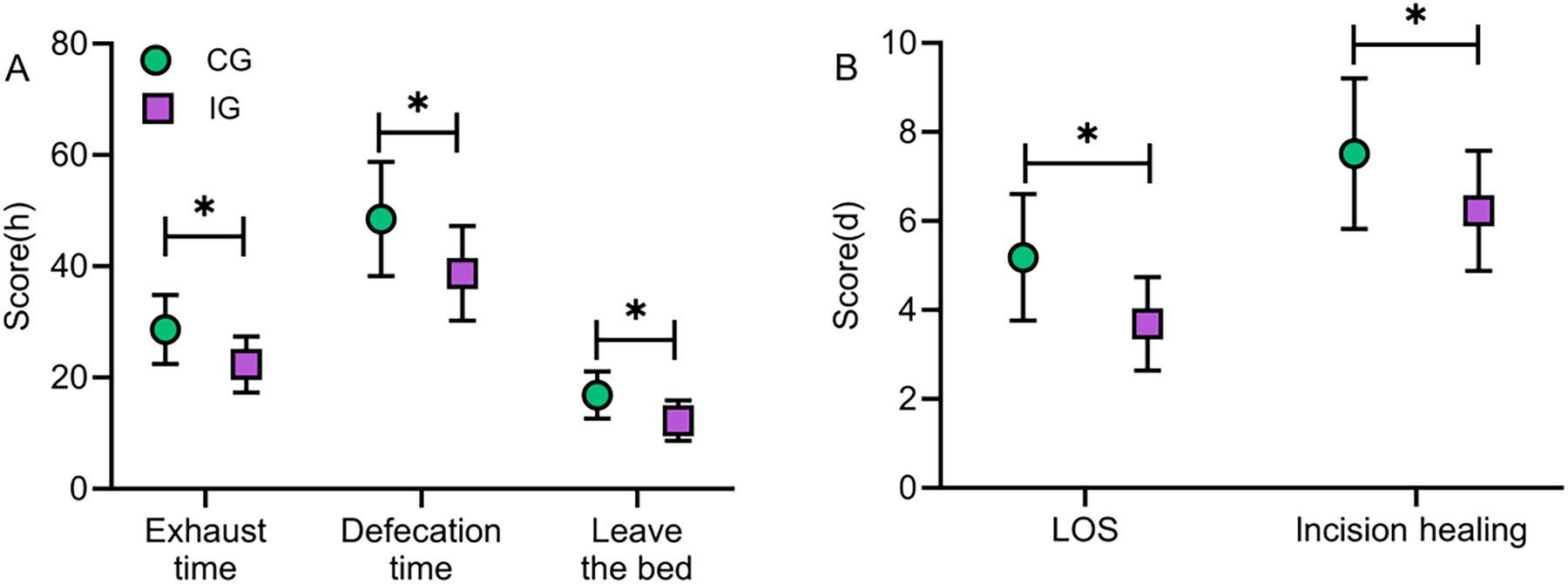
Comparison of postoperative recovery indicators between the two groups. The time to first postoperative flatus, first postoperative defecation, time to ambulation **(A)**, length of hospital stay, and incision healing time **(B)** in the IG were significantly shorter than those in the CG (*P* < 0.05). IG, intervention group; CG, the control group; LOS, length of stay. *indicates statistically significant differences between groups.

Postoperative recovery indicators, such as time to first flatus, time to first defecation, time to ambulation, length of hospital stay, and incision healing time, were collected and compared between the two groups. The results showed that all of these indicators were significantly lower in the IG than in the CG (*P* < 0.05). The findings suggest that the IG demonstrated better postoperative recovery compared to the CG, indicating that multimodal nursing interventions can significantly accelerate the rehabilitation process (Figure 1).

### Comparison of pain intensity between the two groups at different postoperative time points

**Figure 2:**
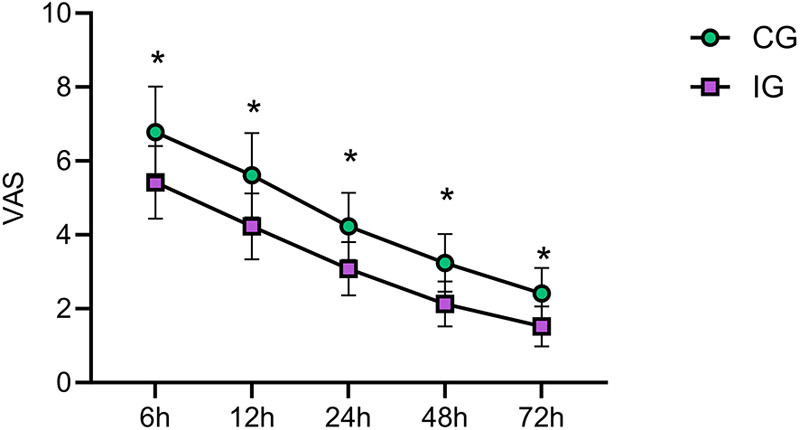
Comparison of pain intensity between the two groups at different postoperative time points. The VAS scores in the IG were lower than those in the CG at 6 h, 12 h, 24 h, 48 h, and 72 h after surgery (*P* < 0.05). IG, intervention group; CG, the control group; VAS, visual analog scale. *indicates statistically significant differences between groups.

Pain intensity in both groups was assessed using the VAS score at 6 h, 12 h, 24 h, 48 h, and 72 h after surgery. Intergroup comparisons were conducted. The results showed that the IG showed lower VAS scores than CG at these observation points (*P* < 0.05). This suggests that the IG achieved superior pain control compared to the CG, indicating that effective perioperative pain management significantly improves patients’ quality of life during the perioperative period and even throughout the rehabilitation phase (Figure 2).

### Comparison of physiological state scores between the two groups

**Figure 3:**
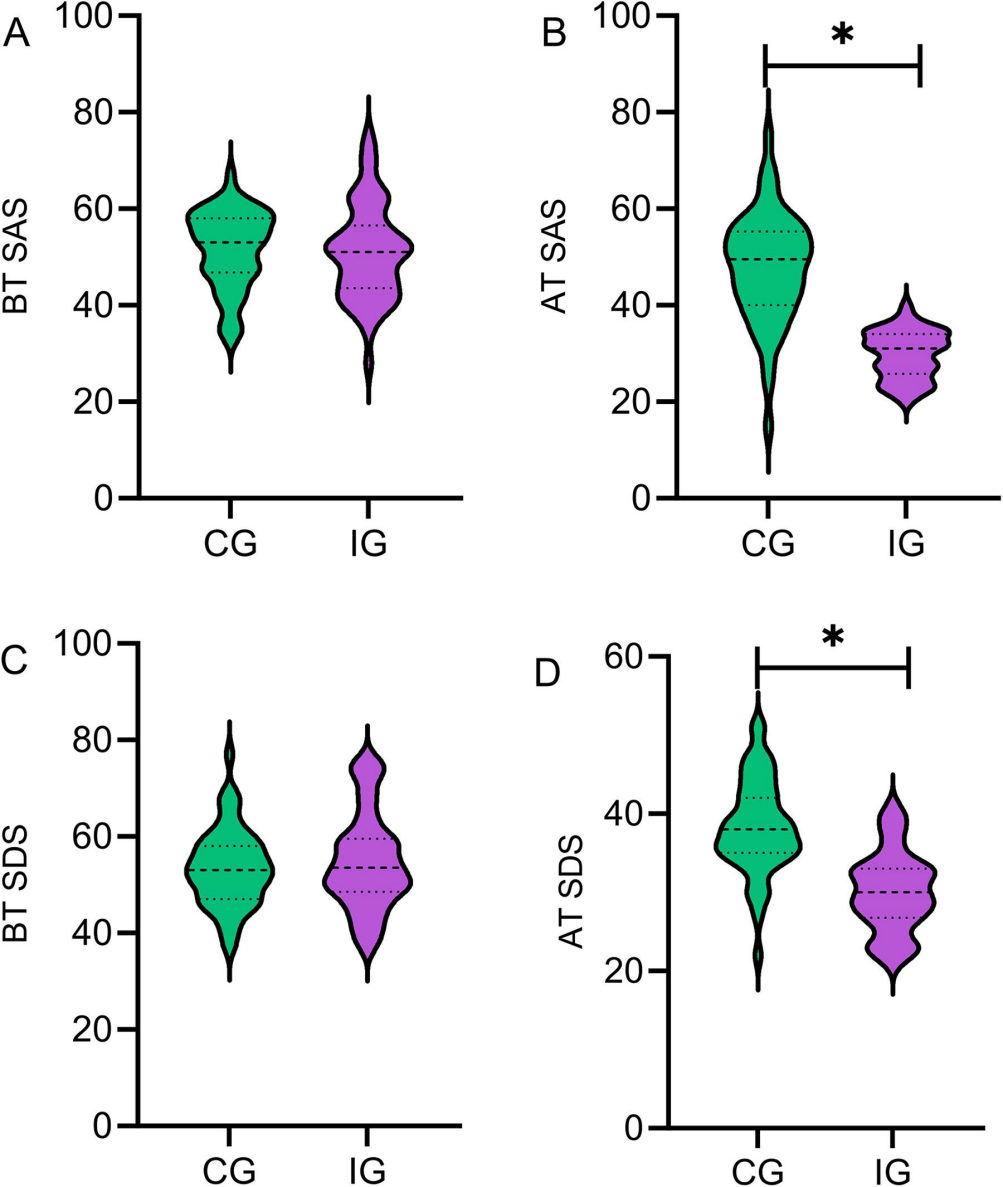
Comparison of physiological state scores between the two groups. Before treatment, there was no statistically significant difference in SAS **(A)** and SDS **(C)** scores between the two groups (*P* > 0.05). At discharge, both SAS **(B)** and SDS **(D)** scores in the IG were significantly lower than those in the CG (*P* < 0.05). IG, intervention group; CG, the control group; BT, before treatment; AT, after discharge. *indicates statistically significant differences between groups.

Preoperatively, there was no statistically significant difference in SAS and SDS scores between the two groups (*P* > 0.05). At discharge, both SAS and SDS scores in the IG were significantly lower than those in the CG (*P* < 0.05). The findings suggest that the intervention was effective in improving patients’ physiological state. The reduction in anxiety and depression scores not only enhances patients’ subjective well-being but may also promote physiological recovery through neuroendocrine-immune pathways (Figure 3).

### Comparison of perioperative immune parameters between the two groups

**Figure 4:**
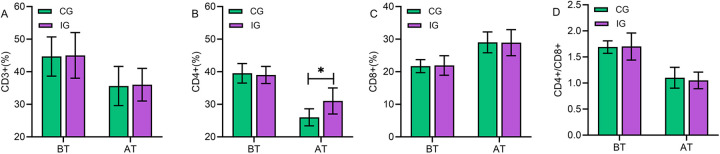
Comparison of perioperative immune parameters between the two groups. There were no statistically significant differences between the two groups in the immune markers CD3+ **(A)**, CD4+ **(B)**, CD8+ **(C)**, and CD4+/CD8+ **(D)** levels at admission (*P* > 0.05). At discharge, the CD4+ level was higher in the IG than in the CG (*P* < 0.05). There were no statistically significant differences in other markers (*P* > 0.05). IG, intervention group; CG, control group; BT, before treatment; AT, at discharge. *indicates statistically significant differences between groups.

Comparison revealed no statistically significant differences in the immune parameters CD3+, CD4+, CD8+, and CD4+/CD8+ levels between the two groups at admission (*P* > 0.05). At discharge, the CD4+ level in the IG was higher than that in the CG (*P* < 0.05). The remaining parameters showed no statistically significant differences (*P* > 0.05). Elevated CD4+ T lymphocyte levels indicate improved immune function, a finding with significant clinical implications. This suggests that multimodal nursing interventions may promote immune recovery by alleviating stress responses and enhancing nutritional and psychological status (Figure 4).

### Comparison of postoperative complications between the two groups

The incidence of various perioperative complications in the two groups was collected using an information system and compared between the two groups. The results showed that the incidence of abdominal distension and the total incidence of complications in the IG were significantly lower than those in the CG (*P* < 0.05). The reduction in the incidence of complications not only improves patient outcomes but also reduces the medical burden and patient suffering, which has significant clinical and health economic implications (Table 2).

**Table 2:** Comparison of postoperative complications between the two groups.

Complication type	Control group (*n* = 50)	Intervention group (*n* = 50)	*χ* ^2^	*P*
Nausea and vomiting	12 (24.00%)	5 (10.00%)	3.383	0.066
Abdominal distension	15 (30.00%)	6 (12.00%)	4.883	0.027
Incision infection	3 (6.00%)	1 (2.00%)	1.042	0.307
Bile leakage	1 (2.00%)	0 (0.00%)	1.010	0.315
Total incidence	18 (36.00%)	8 (16.00%)	5.198	0.023

### Comparison of functional recovery scores between the two groups at discharge

**Figure 5:**
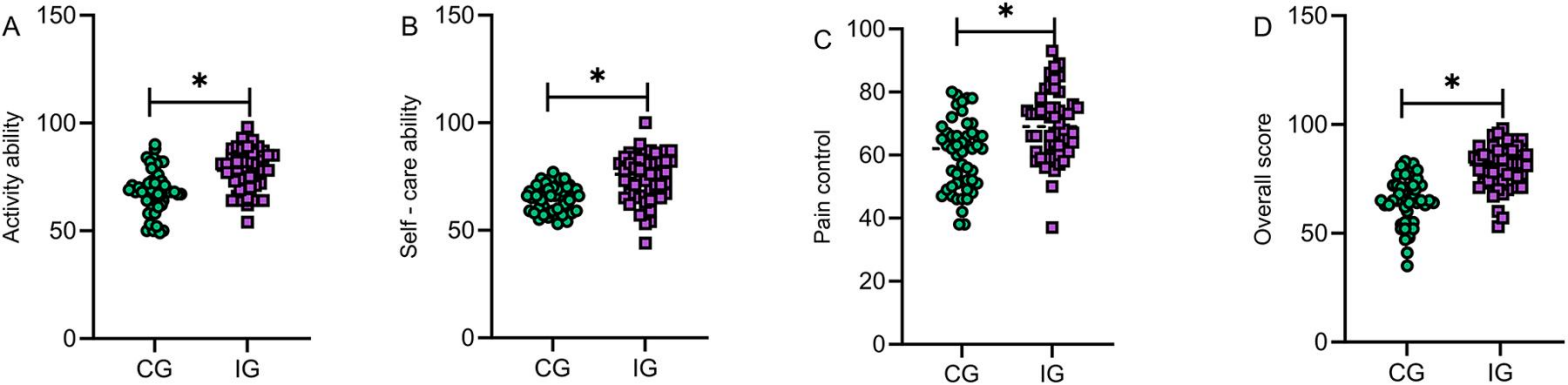
Comparison of functional recovery scores between the two groups at discharge. Patients in the IG had significantly higher scores in daily activities **(A)**, self-care ability **(B)**, pain control **(C)**, and overall recovery **(D)** than those in the CG (*P* > 0.05). IG, intervention group; CG, control group. *indicates statistically significant differences between groups.

The comparison showed that patients in the IG had significantly higher scores in daily activities, self-care ability, pain control, and overall recovery than those in the CG (*P* > 0.05). The comprehensive improvement in functional recovery scores indicates that multimodal nursing interventions can promote patient rehabilitation from multiple dimensions and improve long-term quality of life (Figure 5).

## Discussion

This study systematically evaluated the comprehensive effects of a multimodal nursing intervention combined with health education on patients undergoing LC using a retrospective cohort method. The results showed that this nursing model demonstrated significant advantages in promoting postoperative recovery, controlling pain, improving psychological state, regulating immune function, and reducing complications, providing important evidence-based support for the nursing practice of LC patients. The results of this study are consistent with the conclusions of other international research, confirming the important role of multimodal nursing interventions in perioperative rehabilitation (16).

### Effect of multimodal nursing intervention combined with health education on postoperative recovery

The results of this study showed that patients in the IG significantly outperformed those in the CG group in all recovery indicators, including shortened gastrointestinal function recovery time, early ambulation, and reduced length of hospital stay. This finding is consistent with other related studies (17, 18) and fully demonstrates the effectiveness of the multimodal nursing model. Gastrointestinal function recovery is an important indicator for evaluating recovery after abdominal surgery. The significant shortening of the first flatus and defecation time in the IG was mainly due to the implementation of comprehensive measures such as early mobilization, abdominal massage, and reasonable diets. Specifically, early mobilization can promote intestinal peristalsis, prevent intestinal adhesions, and accelerate the recovery of gastrointestinal function, which is highly consistent with the principle of early mobilization emphasized in the ERAS concept (19). Meanwhile, individualized dietary guidance helps prevent gastrointestinal stimulation caused by premature intake of solid foods, and also avoids the inhibition of gastrointestinal function caused by excessive fasting. From a pathophysiological perspective, early mobilization may accelerate gastrointestinal recovery through mechanisms such as activating the parasympathetic nervous system, promoting gastrointestinal hormone secretion, and improving intestinal blood flow (19, 20). Further comparisons showed that the length of hospital stay of patients in the IG was significantly shorter, which not only reflects the faster recovery of patients, but also has important health economic significance. The reduction in the hospital stay of patients in the IG may reduce medical expenses and the risk of nosocomial infections, which is positive for improving bed turnover rate (21). The comparison of the results in terms of incision healing time may be related to factors such as the improved nutritional status of the patients in the IG, increased activities promoting local blood circulation, and improved psychological state. Several studies have also confirmed that multimodal nursing interventions can shorten hospital stays by 1–2 days and reduce medical costs by 15%–20% (22).

### Optimizing pain management with multimodal nursing intervention and health education

Pain is one of the main factors affecting postoperative recovery after LC. The results of this study showed that the VAS scores of the IG were significantly lower than those of the CG at all postoperative time points, indicating that the multimodal pain management strategy achieved good results. Previous studies (23) have pointed out that good perioperative pain management is important for the overall recovery of patients. The core concept of multimodal analgesia selected in this study is to block the transmission and perception of pain signals at different levels through the combined use of multiple analgesic methods. For example, preventive intervention before pain occurs can reduce the occurrence of central sensitization (24). Non-drug analgesic measures such as music therapy and relaxation training exerts their effects by activating the endogenous analgesic system, diverting attention, and relieving anxiety. Previous studies (25) suggest that although non-drug pain interventions are not effective when used alone, they can produce good synergistic effects when used in combination with drug analgesia. From a neurobiological perspective, music therapy can produce analgesic effects by modulating the limbic system and releasing endorphins, whereas relaxation training alleviates pain by reducing sympathetic nervous system activity and decreasing the release of stress hormones, which is of great significance for improving the long-term quality of life of patients (26). Finally, this study also found that good pain control not only improved the patient's subjective feelings, but also had a positive impact on immune function recovery and complication prevention by reducing stress response, promoting early mobilization, and improving sleep quality. Compared to other research, this study places more emphasis on the importance of preventative analgesia and non-pharmacological interventions.

### Effects of multimodal nursing intervention combined with health education on improving psychological state

Surgery, as a psychological stressor, often leads to the occurrence of negative emotions such as anxiety and depression (27, 28). The results of this study showed that although there was no significant difference in the psychological state of the two groups of patients before surgery, the SAS and SDS scores of the IG were significantly lower than those of the CG at discharge, indicating that this nursing model can effectively improve the psychological state of patients. There are many reasons for the results reported in this study; in other words, psychological nursing intervention may exert its effects through a variety of mechanisms. For instance, cognitive behavioral intervention helps patients establish correct disease cognition and reduce fear caused by misunderstanding; emotional support and therapeutic communication allow patients to feel the care of medical staff and enhance their sense of security and trust; mastering relaxation techniques provides patients with tools for self-regulation; and systematic health education reduces anxiety caused by uncertainty by increasing patients’ knowledge reserves and self-efficacy (29). Through the combined effects of the above intervention measures, on the one hand, the quality of life of patients can be improved, and on the other hand, it can have a positive impact on physiological recovery through the neuro-endocrine-immune network. This has also been confirmed in previous studies. Previous studies (30) found that a positive physiological state helps regulate stress hormone levels, improve immune function, and promote wound healing. From a psychoneuroimmunology perspective, improvements in psychological state can regulate cortisol levels through the hypothalamic-pituitary-adrenal (HPA) axis, thereby influencing T lymphocyte function. This finding aligns with other studies, which confirm that perioperative psychological interventions can reduce the incidence of postoperative anxiety (31).

### Regulatory effect of multimodal nursing intervention combined with health education on immune function

This study innovatively incorporated the assessment of immune function indicators. The results showed that CD4+ levels in the IG were significantly higher than those in the CG at discharge. CD4+ T lymphocytes, as helper T cells, play a central role in both cellular and humoral immunity. Increased levels of CD4+ T lymphocytes indicate improved immune function. Previous studies (32) have pointed out that surgery, as a strong stressor, can lead to significant immunosuppression in the body, which is manifested in a decrease in the number of T lymphocytes and a decline in their function. The reasons for the improvement in CD4+ levels in the IG in this study may be as follows: (1) Effective pain control reduces the suppression of the immune system by stress response; (2) Adequate sleep and nutritional support provide a basis for the proliferation and functional maintenance of immune cells; (3) Early mobilization improves the function of the circulatory system, which is beneficial to the distribution and function of immune cells; (4) The improvement of psychological state has a positive effect on immune function through neuroendocrine pathways. From an immunological perspective, the elevation of cortisol induced by surgical stress inhibits the production of Th1 cytokines (such as IL-2 and IFN-γ), thereby affecting the activation and proliferation of CD4+ *T* cells, whereas multimodal nursing interventions may reverse this process by alleviating the stress response (31, 33). Finally, although the inter-group differences in other immune indicators (CD3+, CD8+, CD4+/CD8+) did not reach statistical significance, the IG showed an improvement trend, which may be related to factors such as sample size and observation time. This further emphasizes the necessity and importance of conducting prospective studies in the future.

### Effect of multimodal nursing intervention combined with health education on the prevention of complications

This study showed that the overall incidence of postoperative complications in the IG was significantly lower than that in the CG, with a particularly significant reduction in the incidence of abdominal distension. This finding is of great clinical significance. It has been found that abdominal distension is one of the most common discomfort symptoms after LC surgery, which is mainly related to factors such as residual CO_2_ pneumoperitoneum, gastrointestinal dysfunction, and reduced activity (34). The IG promoted the excretion of residual CO_2_ through early mobilization, avoided gas-producing foods through reasonable dietary guidance, and promoted intestinal peristalsis through abdominal massage. These multiple measures effectively reduced the occurrence of abdominal distension. In addition, the health education conducted in advance allowed patients to understand the causes and prevention methods of abdominal distension, which improved their cooperation. This may also be one of the important reasons for the reduced incidence of abdominal distension in the IG. Although the incidence of nausea and vomiting did not reach statistical significance, the IG still showed a decreasing trend, which may be related to factors such as effective pain control reducing the use of opioids and psychological intervention alleviating anxiety. The incidence of incision infection and bile leakage was low, which may be related to the limited sample size. However, the IG theoretically helped reduce the risk of these complications through measures such as strengthening nutritional support and improving immune function. From the perspectives of patient safety and healthcare costs, the reduction of complications can significantly reduce readmission rates and medical expenses, thereby improving the safety of perioperative management. Furthermore, the reduction in the incidence of complications can also significantly improve patient satisfaction with clinical interventions.

### Comprehensive impact of multimodal nursing intervention combined with health education on functional recovery

Functional recovery scores comprehensively reflect the quality of patient rehabilitation from four dimensions: activities of daily living, self-care ability, pain control, and overall recovery. The IG scored significantly higher than the CG in all dimensions, indicating that this nursing model can comprehensively improve patients’ functional recovery levels. This comprehensive functional improvement is the result of the combined effect of multiple factors: effective pain control creates conditions for early mobilization; systematic rehabilitation exercises promote the recovery of motor function; a good physiological state enhances the initiative of rehabilitation; and adequate health education improves self-management ability. The above factors promote each other, forming a virtuous circle and ultimately achieving the optimization of functional recovery. From a long-term prognosis perspective, improvements in functional recovery are not only reflected during hospitalization but may also continue after discharge, affecting the patient's work ability and quality of life.

### Research innovation, limitations, and future prospects

This study is innovative in incorporating immune function indicators into the nursing effectiveness evaluation system for LC patients. This multidimensional evaluation index comprehensively reflects the value of multimodal nursing interventions. Multidimensional evaluation is a crucial means of implementing the overall concept of nursing practice and is an important lever for improving the comprehensive indicators of patients in the perioperative period. This study provides a detailed description of multimodal nursing interventions, providing a reference for other research. Additionally, this study is the first to systematically evaluate the impact of multimodal nursing interventions on immune function in patients with LC, filling a research gap in this field and providing new evidence to support the application of the ERAS concept in nursing practice. This study also has several limitations. (1) The retrospective design may be susceptible to information bias and selection bias, and its control of confounding factors is less thorough than that in prospective studies. (2) The single-center design of this study limited the generalizability of the results, as nursing levels and patient characteristics may differ across medical institutions. (3) The relatively small sample size affected the ability to detect complications with low incidence rates. (4) The limited follow-up period prevented the assessment of long-term outcomes. (5) A cost-effectiveness analysis was not conducted, and the health economic value of this nursing model was not evaluated. In view of the above limitations, future research will focus on increasing the sample size and conducting multicenter prospective studies to further explore the feasibility of individualized nursing strategies.

In summary, multimodal nursing intervention combined with health education has achieved significant clinical results by integrating multiple nursing strategies to provide comprehensive intervention for patients undergoing laparoscopic cholecystectomy from multiple levels, including physiological, psychological, and immune aspects. This nursing model can not only promote postoperative recovery, optimize pain management, and improve psychological state, but also regulate immune function, reduce the incidence of complications, and improve the quality of functional recovery. This patient-centered nursing concept that focuses on integrity and individualization is in line with the development direction of modern nursing and is worthy of promotion and application in clinical practice.

## Data Availability

The original contributions presented in the study are included in the article/Supplementary Material, further inquiries can be directed to the corresponding author.
